# Comment on “Lack of Association Found between* Helicobacter pylori* Infection and Diarrhea-Predominant Irritable Bowel Syndrome: A Multicenter Retrospective Study”

**DOI:** 10.1155/2016/6148927

**Published:** 2016-10-11

**Authors:** Amin Talebi Bezmin Abadi

**Affiliations:** Department of Bacteriology, Faculty of Medical Sciences, Tarbiat Modares University, Tehran, Iran

We have read with great interest the paper written by Feng Xiong et al. “Lack of Association Found between* Helicobacter pylori* Infection and Diarrhea-Predominant Irritable Bowel Syndrome: A Multicenter Retrospective Study” [1]. From the beginning, possible association between* H. pylori* infection and diarrhea-predominant irritable bowel syndrome (IBS-D) was a challenging topic among clinicians [2]. In this paper, authors concluded that no significant correlation between* H. pylori* infection and IBS-D was reported. Moreover, IBS-D patients may not benefit from* H. pylori* eradication. Apart from being an interesting study, there is a short list of critical points to draw a better conclusion within such multicenter studies worldwide. (1) Sample size is mainly lacking in this study. Relatively high rate of treatment efficacy observed in* H. pylori* infected group can be bound to the low number of investigated individuals. (2) Lack of long-term follow-up in this study is leading to incorrect conclusion regarding relieving bloating symptoms. Moreover, insufficient study design for treatment of the bacterium was another pitfall causing not strong conclusion. According to the current findings, authors concluded that IBS-D patients do not benefit from* H. pylori* eradication. As a note, determination of successful* H. pylori* eradication is not clearly described and validated in this study. Also, we need a long-term follow-up (at least 6 months) to confirm primary conclusion drawn by the authors. (3) With regard to the current findings, to be honest, it seems that we have a lack of better evidence rather than lack of association. In conclusion, we agree with Xiong et al., if proven in further studies, about the underlying mechanisms determining* H. pylori* role in IBS. Finally, the understanding of the* H. pylori*-host interaction in IBS subjects may open the insight into the development of new therapeutic interventions to manage* H. pylori* associated extra-digestive disease.
